# The 50th ASMS Conference on Mass Spectrometry and Allied Topics

**DOI:** 10.1002/cfg.207

**Published:** 2002-10

**Authors:** Francesco L. Brancia

**Affiliations:** 1 Shimadzu Research Laboratory (Europe) Wharfside Trafford Wharf Road Manchester M17 1GP UK

## Abstract

Development of new mass spectrometers and implementation of new analytical methods were the central themes of the conference. The majority of oral presentations and posters were concerned with the application of mass spectrometry to pharmaceutical and biotechnological research.


**Comparative and Functional Genomics**
*Comp Funct Genom* 2002; **3**: 455–458.
Published online in Wiley InterScience (www.interscience.wiley.com). **DOI:** 10.1002/cfg.207

**Feature**

### Meeting Review: the 50th ASMS conference on mass spectrometry and allied topics

Orlando, Florida, USA, 2–6 June 2002

Francesco L. Brancia*
*Shimadzu Research Laboratory (Europe), Wharfside, Trafford Wharf Road, Manchester M17 1GP, UK*

**Correspondence to:
Francesco L. Brancia, Shimadzu Research Laboratory, Wharfside, Trafford Wharf Road, Manchester M17 1GP, UK.
E-mail:
Francesco.brancia@srlab.co.uk*

Received: 18 July 2002
Revised: 31 July 2002

### Abstract

**Development of new mass spectrometers and implementation of new analytical methods were the central themes of the conference. The majority of oral presentations and posters were concerned with the application of mass spectrometry to pharmaceutical and biotechnological research. Copyright © 2002 John Wiley & Sons, Ltd.**

On the occasion of the 50th anniversary of the American Society for Mass Spectrometry, the annual conference was opened by a plenary lecture given by R. Graham Cooks (Purdue University, West Lafayette, IN, USA). In his presentation, entitled 'Historical Perspectives of Mass Spectrometry', he reviewed the history of mass spectrometry from the first mass spectrum obtained by J. J. Thomson until the most recent applications in the biological field. Since its invention, mass spectrometry has become the most versatile analytical method and it has been utilized to enhance our knowledge in physics, chemistry, surface science, earth and planetary science, molecular biology and medicine. Recently becoming more than a tool, mass spectrometry has emerged as a separate discipline — the science of gas-phase ions — with an intrinsic subject matter and conceptual basis. His list of the 10 most important mass spectra contained milestones that have changed the face of modern mass spectrometry. Among these, it is worth noting the fast atom bombardment (FAB) spectrum of insulin, the first matrix-assisted laser desorption ionization (MALDI) spectrum, the electrospray (ES) spectrum of polyethylene glycol and the first spectrum containing evidence of charge-remote fragmentation.

### Proteomics: intact protein characterization

Most of the mass spectrometry (MS)-based methods for protein identification rely upon a strategy involving proteolytic digestion of the protein mixtures. Subsequent mass spectrometric analysis employs peptide mass fingerprinting (PMF) and/or tandem mass spectrometry (MS–MS) of the ion of interest. This approach is also referred to as the 'bottom-up approach' for protein identification. Although this method is widely used, several aspects can compromise its implementation. The high complexity of the peptide mixtures and the limited ability of the chromatographic techniques to completely separate the mixtures into all components are the main limitations of the 'bottom-up' approach. As a result, discrimination effects towards low molecular weight proteins exist and they are detrimental to the identification of protein mixtures.

By contrast, the 'top-down' approach to protein identification consists of gas-phase dissociation of

Copyright © 2002 John Wiley & Sons, Ltd.

intact protein ions without the help of proteolytic digestion prior to mass spectrometric analysis. To date, two complementary routes have been proposed for protein identification in the 'top-down' approach. The first method is based upon Fourier transform ion cyclotron resonance mass spectrometry (FTICR–MS), in which tandem mass spectrometry is performed by using electron capture dissociation (ECD) as the activation technique. In the talk, entitled 'Top-down characterization of proteins by electron capture dissociation mass spectrometry: an application to the proteome of *Mycobacterium tuberculosis*', Ying Ge (Cornell University, Ithaca, NY, USA) utilized this methodology to study the proteins secreted by *Mycobacterium tuberculosis* (MTB) that are essential to the pathogenesis of this organism. The protein mixtures precipitated under different conditions are directly infused into the mass spectrometer to generate an ES spectrum in which all components are visible due to the high resolving power of the mass spectrometer. After isolation of the most abundant ions, MS–MS analysis was performed, providing structural information utilized for protein identification. Although 10 proteins were identified directly from protein mixtures by sequence tag searching, only two were novel secreted proteins. Due to the intrinsic nature of the ECD process, post-translational modifications are preserved and secondary and tertiary structure of proteins can be studied. After enrichment on a ConA Sepharose column, five secreted glycoproteins containing up to seven hexose molecules were observed in an ES spectrum.

The alternative 'top-down' strategy involves ES MS–MS of the intact protein in low-resolution instruments (quadrupole ion trap). To facilitate interpretation of the production spectrum, the entire product ion population is subjected to gas-phase ion–ion proton transfer with singly-charged anions, in the time scale of milliseconds, by using atmospheric sampling glow-discharge ionization (ASGDI). The ion–ion reaction experiment results in a reduction of the charge state of the multiply charged product ions. Singly-charged ions dominate the resulting MS–MS spectrum, facilitating data interpretation. In the oral presentation entitled 'Gas-phase concentration, purification and identification of whole proteins from complex mixtures', Gavin E. Reid (Purdue University, West Lafayette, IN, USA) provided further evidence for the validity of this method. In conjunction with 'ion parking', ion–ion reactions can be used in a quadrupole ion trap to selectively concentrate protein ions that are initially dispersed over a range of charge states, into a single charge-state. The methodology was applied to the analysis of a complex mixture containing approximately 30 proteins derived from a whole cell lysate fraction of *Escherichia coli*. Five of the most abundant protein components were concentrated and purified in gas phase prior to collisionally activated dissociation (CAD). Subsequent protein identification was performed by matching the unknown product ion spectra against a partially annotated protein sequence database.

### Quantification of protein expression

In protein-expression mapping, the ability to quantify alterations in protein abundance resulting from internal or external perturbations of the cell is crucial to the functional analysis of biological systems. In recent years, many efforts have been devoted to developing chemical strategies for relative quantitative analysis of proteins in complex mixtures. The common strategy involves derivatization of each set of proteins/peptides with isotopical variants of the same chemical reagent/label. The samples are then combined and differentiated, using mass spectrometry after purification. This approach is based upon the assumption that the two sets of isotopically labelled peptides behave identically throughout sample manipulation and mass spectrometric analysis. In his poster entitled 'Improved characterization and quantification of proteins using a novel multipurpose chemical derivatisation', Shabaz Mohammed (UMIST, Manchester, UK) presented an innovative method for relative quantification. The methodology relies on a procedure (guanidination) that converts lysine into homoarginine residues using *O*-methylisourea. This has the effect of improving the signal intensities of the lysine-containing peptides and increases the protein sequence coverage in MALDI spectra. In the new development of this methodology, the labelling reagent *O*-methylisourea was synthesized in both isotopical variants, incorporating either $^{14}N$ or $^{15}N$. Using a typical relative protocol, the validity of the method was demonstrated using a mixture of equal portions of a digest of yeast enolase derivatized with [$^{14}N$] and [$^{15}N$] *O*-methylisourea. The MALDI spectrum contains

Copyright © 2002 John Wiley & Sons, Ltd.

doublets corresponding to peaks associated with the lysine tryptic peptides. From the relative intensities of the two ion signals it is possible to derive the relative abundance of one protein against another. The method has additional advantages for peptide sequencing. When peptide mixtures incorporating both isotopic variants are analysed by tandem mass spectrometry, the y ions (containing the modification at C-terminus) appear as doublets. The advantages of this approach are robustness, low cost and simplicity of the chemistry involved. The main limitation of this approach is that quantification is relative and it does not measure gene expression at the level of functional protein.

In his presentation entitled 'Absolute quantification of cell cycle regulatory proteins and phosphorylation states: the AQUA strategy for protein profiling', Scott A. Gerber (Harvard Medical School, Boston, MA, USA) presented a new method called AQUA to detect and accurately measure both protein expression and protein phosphorylation on a absolute scale, directly from whole-cell lysates. The approach relies on the use of isotopically labelled proteolytic peptides as internal standards. In order to minimize interference during the chromatographic analysis, peptides are chosen according to their chemical (hydrophobicity, retention times, etc.) and biological features (low amino acid similarity with other peptides). A defined quantity of isotopically labelled peptides is diluted in the whole cell lysate. The quantitative approach is performed by selective reaction monitoring (SRM) analysis for synthetic and native peptides after tryptic digestion from the whole cell lysate on a triple quadrupole mass spectrometer. Assuming that the electrospray response for both peptides is identical, it is possible to correlate response of the *in vivo* peptide vs. ion intensity of the synthetic peptide. As a demonstration of this strategy, the human protein separase was studied in both its phosphorylated and non-phosphorylated state during the cell cycle. Although the AQUA method provides absolute quantification of the protein of interest without using any chemical derivatization, it is labour-intensive and time-consuming.

### Instrument developments

Marvin Vestal (Applied Biosystems, Framingham, MA, USA) presented the current state of time-of-flight (ToF) mass spectrometry in a talk entitled 'The future of time-of-flight mass spectrometry'. Developments such as delayed extraction with MALDI and orthogonal injection with electrospray ionization have provided resolving power and mass accuracy of time-of-flight analysers that is superior to that available with quadrupoles and quadrupole traps, and competitive with that from more expensive techniques, such as magnetic deflection and FTICR–MS. In the future, dramatic improvements in the speed and utility of ToF are expected. In ToF it is theoretically possible to generate more than 10 000 complete mass spectra/s over an essentially unlimited mass range, but in most practical applications the number of ions in each such spectrum is insufficient to provide the required precision of mass or abundance measurement. At the moment, recorded spectra are generally the sum of a number of individual spectra, and existing data systems can only record and process these spectra at rates on the order of 1 spectrum/s. Recent developments in instrumentation and computers suggest that in the near future high quality MS and MS–MS spectra can be generated, recorded and interpreted at rates in excess of 100 spectra/s.

Emmanuel Raptakis (Kratos Analytical, Manchester, UK) gave an oral presentation entitled 'Experimental study of mass accuracy and mass resolution in a MALDI quadrupole ion trap (QIT) time-of-flight (ToF) mass spectrometer'. Spectra displaying the high accuracy (either in normal MS or MS–MS mode) of a new hybrid mass spectrometer were shown. This instrument comprises a quadrupole ion trap (QIT) and a time-of-flight mass (ToF) analyser. The three-dimensional device has two functions: first, it traps externally generated MALDI ions; second, it isolates the ion of interest by ejecting the rest of ions from the storing device. The selected ion population is then activated by collision with an inert gas (Ar), producing a range of fragment ions that can provide information on the structure of the selected product ion. The application of time-of-flight technology provides a high mass resolution, either in MS or in tandem mass spectrometric analysis. Selecting an ion with resolution bigger than 1000, it is possible to isolate one single isotope for product ion scanning, excluding all potential contaminant peaks. Additionally, with a mass accuracy less than 10 ppm, a single calibration can be used across all modes of analysis.

Copyright © 2002 John Wiley & Sons, Ltd. 

The possibility of reducing the error window on experimental peptide mass values allows improvements in the confidence of protein identification by database searching. The key feature of the instrument is the ability to perform multiple tandem mass spectrometric analyses up to $MS^4$. After decomposition of the precursor ion, a resulting product fragment ion can be isolated and dissociated again. This multiple procedure allows observation of immonium ions and produces structural information beneficial to correct protein identification.

In the 'Tomorrow's MS Hardware' session, James W. Hager (MDS SCIEX, Concord, Canada) described a new linear ion trap mass spectrometer in his talk, 'Performance of a hybrid RF/DC quadrupole-linear ion trap mass spectrometer'. The presentation showed the applications of a linear ion trap that uses mass selective axial ion ejection. The instrument is based on a triple quadrupole mass spectrometer in which either the collision cell or the final resolving quadrupole were converted into a linear ion trap device. An ion injection efficiency approaching 100% for injection of low-energy ions was reported, together with about 20% extraction efficiency. The novelty of this configuration is that modification of a triple quadrupole mass spectrometer incorporating a linear ion trap with axial ion ejection can improve MS–MS performance while maintaining all of the triple quadrupole capabilities, such as the ability to carry out precursor ion and neutral loss scanning and multiple reaction product monitoring with no performance loss.

### Bioinformatics

Efforts have been devoted to the development of new software for *de novo* sequencing. Bin Ma (University of Western Ontario, London, ON, Canada) presented a talk entitled 'A powerful software tool for *de novo* sequencing of peptides from MS–MS data'. Nowadays, the concept of using non-database-dependent software for interpretation of MS–MS data is becoming increasingly popular. Instead of using a database searching algorithm, in which the ion signals are compared to the theoretical data obtained applying certain rules of cleavage to the 'virtual' proteome (specificity of the proteolytic enzyme for PMF, fragmentation of peptide backbone with formation of a/b/c/x/y/z ion series for MS–MS analysis), the *de novo* software tries to interpret the data without the advice of any pre-existing dataset. In this approach, a matching score between peptide and observed spectrum is defined first. The problem, then, is to find the peptide with the highest matching score with respect to the observed spectrum. In order to obtain a list of possible candidates, the rules of cleavage generating a/b/c/x/y/z fragment ions are applied to interpret the MS–MS data. According to the score obtained, peptide candidates are ranked, providing the hit with the highest score.

### Conclusion

With the advent of the proteomics era, the demand for new techniques for identification and quantification of proteins has greatly increased and a large proportion of the mass-spectrometry community is focused on developing new MS instruments, chemical methods and bioinformatics tools to study the protein networks of different organisms. In fact, the conference was dominated by oral presentations and scientific posters aimed at the analysis of proteomes.

---

The Meeting Reviews of Comparative and Functional Genomics aim to present a commentary on the topical issues in genomics studies presented at a conference. The Meeting Reviews are invited; they represent personal critical analyses of the current reports and aim at providing implications for future genomics studies.

---

Copyright © 2002 John Wiley & Sons, Ltd. 

